# Investigating the impact of metformin on severity of COVID‐19 in patients with Type 2 diabetes mellitus: Focusing on laboratory findings

**DOI:** 10.1002/edm2.441

**Published:** 2023-07-11

**Authors:** Rana Taheri, Seyedeh Zahra Shahrokhi, Zahra Amjadi, Faranak Kazerouni

**Affiliations:** ^1^ Department of Clinical Biochemistry, School of Medicine Shahid Beheshti University of Medical Sciences Tehran Iran; ^2^ Division of Biochemistry Fardis Central Lab Alborz Iran; ^3^ Department of Biochemistry, School of Medicine AJA University of Medical Sciences Tehran Iran; ^4^ Department of Biology and Biochemistry, Science Faculty, Shahr‐e‐Qods Branch Islamic Azad University Tehran Iran; ^5^ Department of Medical lab. Sciences, School of Allied Medical Sciences Shahid Beheshti University of Medical Sciences Tehran Iran

**Keywords:** COVID‐19, laboratory parameters, metformin, T2DM

## Abstract

**Background:**

In the terrifying pandemic caused by SARS‐CoV‐2, diabetic patients exhibiting more severe outcomes and mortality rate is high among them. Based on recent studies, metformin as the most prescribed drug for T2DM treatment may improve severe outcomes in diabetic patients infected with SARS‐CoV‐2. On the other hand, abnormal laboratory findings can help to differentiate between the severe and non‐severe form of COVID‐19. According to the mentioned issues, the effect of metformin on severity of COVID‐19 was examined in T2DM patients with SARS‐CoV‐2 infection.

**Methods:**

The study included 187 individuals diagnosed with COVID‐19, 104 patients were diabetic and divided into two groups according to their anti‐diabetic drugs: patients who were treated only with metformin and patients who were treated with other anti‐diabetic drugs. The other participants were non‐diabetic and diagnosed with COVID‐19. Biochemical parameters were measured by routine laboratory methods before, during and after SARS‐CoV‐2 infection.

**Results:**

During infection, FBS, creatinine, ALT, AST, Ferritin and LDH were significantly lower in metformin users than non‐users (*p*‐*value*: .02, .01, .03, .04, .0009 and .01, respectively). Also, after recovery, there were statistically significant differences between metformin users and non‐users with respect to most of the study parameters, except FBS, BUN and ALP (*p‐value*: .51, .28 and .35, respectively).

**Conclusion:**

Our result suggested that metformin might be associated with better outcomes in diabetic patients infected with SARS‐CoV‐2.

## Graphical abstract

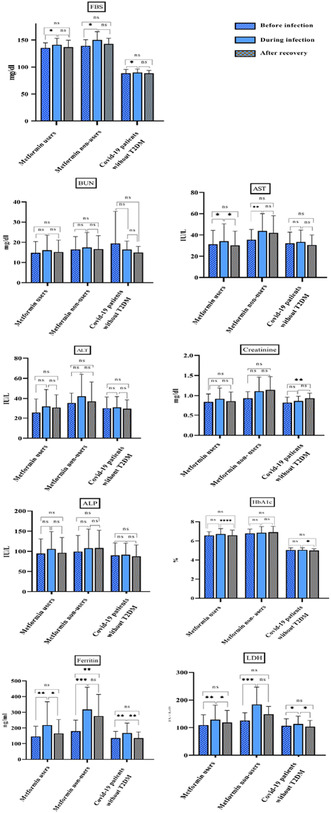

## 1 INTRODUCTION

A novel positive‐stranded RNA virus that causes coronavirus disease 2019 (COVID‐19) has become the most significant global health issue, affecting over 500 billion people and causing over 6 million deaths.^1^ As a systemic syndrome, COVID‐19 patients have a wide range of symptoms that in some cases lead to hospitalisation. Considering this critical situation, except for the clinical features and imaging findings of COVID‐19, identification of laboratory parameters that have a prognostic value is needed.^2^

Numerous retrospective studies have shown abnormal liver chemistries such as elevated levels of alanine aminotransferase (ALT), aspartate aminotransferase (AST) and lactate dehydrogenase (LDH) in patients with COVID‐19, perhaps due to direct liver injury, inflammatory responses and muscle breakdown.^3^

In addition, since the beginning of this cruel disease, studies have determined poor prognostic markers, such as older age, obesity, hypertension, dyslipidaemia, cardiovascular diseases and diabetes, which are associated with severe forms of COVID‐19.^4^ Various studies have shown the relation between diabetes and higher rates of hospitalisation, ICU admission and mortality of COVID‐19. It seems that in the presence of diabetes, the mortality rate of COVID‐19 is double.^5^

Type 2 diabetes mellitus (T2DM) is a chronic metabolic disease characterized by disturbed glucose homeostasis. Unarguably, hyperglycaemia impacts the body's ability to regulate immune and inflammatory responses in individuals with diabetes.^6^ On the other hand, COVID‐19 infection exacerbates hyperglycaemia through affecting pancreatic beta cells and insulin target tissues, thus controlling blood glucose levels is necessary for diabetic patients.^7^ A recent study on diabetic patients infected with coronavirus indicated that appropriate blood glucose control decreased the risk of death by approximately sevenfold.^8^

The most common drug prescribed for lowering blood glucose levels is metformin. Interestingly, besides its glucose lowering potential, metformin can exert various protective effects against inflammation and cardiovascular damage through the activation of AMP‐activated protein kinase (AMPK), endothelial nitric oxide synthase (eNOS) and Sirt1.^9^ Activation of AMPK as a key mediator of metformin has beneficial effects such as phosphorylation (Ser680) of ACE2 receptor which decreases viral entry into the cell and elevates expression and stability of ACE2 that confers pulmonary protection.^10^, ^11^ Furthermore, retrospective studies indicate the relation between metformin treatment and reduced mortality among diabetic patients with COVID‐19.^12^

Given these facts, while diabetes is an independent risk factor for mortality from COVID‐19, it seems that mentioned risk is significantly reduced in people taking metformin, improving the possibility that metformin may play a protective role in this high‐risk population. On the other hand, laboratory tests are used to evaluate the improvement process in COVID‐19 patients.^2^, ^4^ Thus, in the current study, common biochemical factors were compared in T2DM patients (metformin consumer and a group using agents other than metformin), and non‐diabetic COVID‐19 patients. The results of the present study may display the importance of laboratory tests in COVID‐19 patients' follow‐up and shed light on the effect of metformin in controlling infection outcomes in diabetic people.

## 2 MATERIALS AND METHODS

### 2.1 Study design and patient selection

All study steps were performed in compliance with the principles of the Helsinki Declaration, and ethical clearance was approved by the ethics committee at Shahid Beheshti University of Medical Sciences (IR.SBMU.RETECH.REC.1400.1159). All included patients provided informed consent. A total of 187 individuals diagnosed with COVID‐19 were enrolled in our study. All participants had a positive PCR 1–2 days after symptoms manifestation. Among them, 104 patients were diabetic and divided into two groups according to their anti‐diabetic drugs: patients who were treated only with metformin (1000–1500 mg/day), and patients who used drugs other than metformin. Diabetes duration in patients was approximately 2 years and all the patients received their drugs during infection. Participants with a previous history of renal and liver diseases, COVID‐19 vaccination and pregnant women were not included in our study. We compared biochemical parameters at least 3 months before infection, during infection and at least 3–4 months after patients recovered from the disease.

### 2.2 Biochemical tests

In this follow‐up study, biochemical parameters including AST, ALT, ALP, creatinine, BUN, FBS, Ferritin and LDH were analysed by routine laboratory methods before infection, during COVID‐19 infection and after recovery. HbA1c values were quantified using HPLC method. Lab values and corresponding reference ranges were collected from the laboratory information system.

### 2.3 Statistical analyses

We used GraphPad Prism (version 8.4.3) software to perform statistical analysis. The normality of the distributions of variables was carried out using Kolmogorov–Smirnov test. Continuous data were expressed as mean ± SD. An Independent *t*‐test and repeated measures one‐way ANOVA were performed to compare parametric variables. Comparisons between nonparametric variables were made by Friedman and Mann–Whitney *U* test. In the current study, two‐tailed *p*‐values less than .05 were considered statistically significant.

## 3 RESULTS

### 3.1 General characteristics of patients

Our study included 84 COVID‐19 patients without diabetes and 103 T2DM patients with COVID‐19 (52 metformin users and 51 non‐users) which did not differ significantly in age (50.70 ± 5.14 vs. 50.93 ± 5.85, *p*‐value = .86). Among 187 patients, 97 were female and 90 participants were male.

### 3.2 Overall changes in biochemical parameters before infection, during infection and after recovery in study groups

**FIGURE 1:**
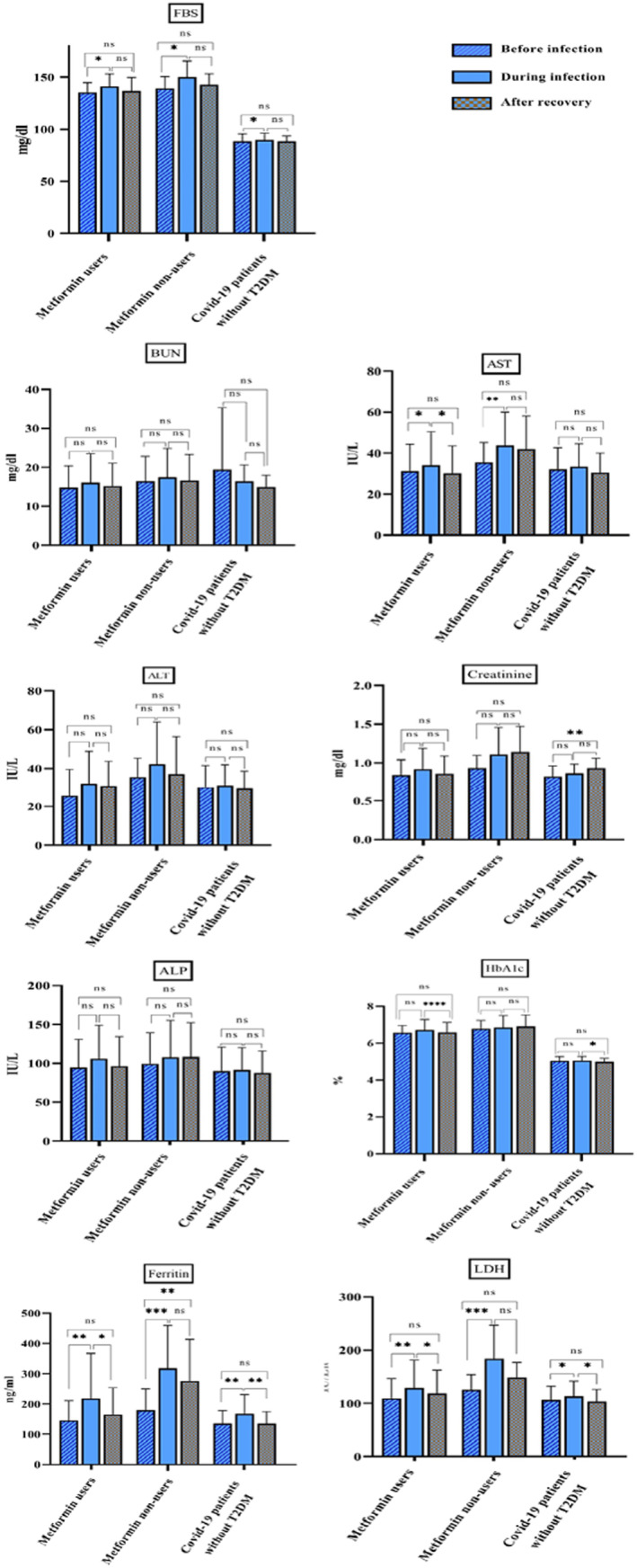
Overall changes in biochemical parameters before infection, during infection and after recovery in study groups.

In all the three groups, FBS was significantly changed during infection (*p*‐value: .019 for metformin users, .005 for metformin non‐users and .04 for COVID‐19 patients without T2DM). Based on the results represented in Figure 1, HbA1c levels were decreased after recovery in metformin users (*p*‐value: <.0001) and individuals without T2DM (*p*‐value: .02). Creatinine levels were reduced after recovery compared with before infection in people without T2DM. Comparison of AST showed that there was a statistically significant difference before disease and during infection in diabetic group (*p*‐value: .04 for metformin users and .006 for other diabetic group). There is also a significant reduction of AST in metformin consumers after recovery (*p*‐value: .03). LDH amounts were increased during infection in all subgroups (*p*‐value: .006 for metformin users, .0009 for non‐users and .04 for people without T2DM). There was also a significant decrease in LDH level after recovery in metformin consumers and healthy people (*p*‐value: .03 and .016, respectively). In metformin users and individuals without T2DM, ferritin levels were higher than before infection (*p*‐value: .009 and .008, respectively) and decreased after recovery (*p*‐value: .03 and .006, respectively). In T2DM patients that use anti‐diabetic agents other than metformin, there was statistically significant difference between before infection and during infection (*p*‐value: .0004) and after recovery (*p*‐value: .003) (Figure 1).

### 3.3 Comparison of biochemical parameters in study groups before infection, during infection and after recovery

#### 3.3.1 Metformin users and metformin non‐users

As shown in Table 1, before COVID‐19 infection, there were no statistically significant differences in biochemical parameters between metformin users and non‐users. During infection, FBS, creatinine, ALT, AST, Ferritin and LDH were significantly lower in metformin‐users than non‐users (*p*‐value: .02, 0.01, .03, .04, .0009 and .01, respectively). After recovery, there were statistically significant differences between metformin users and non‐users with respect to most of the study parameters, except FBS, BUN and ALP.

**TABLE 1:** Comparison of study parameters in metformin users and non‐users before infection, during infection and after recovery.

Before infection				During infection			After recovery		
Variables	Metformin users	Metformin non‐users	*p*‐Value	Metformin users	Metformin non‐users	*p*‐Value	Metformin users	Metformin non‐users	*p*‐Value
FBS (mg/dl)	135.7 ± 9.30	138.5 ± 11.54	.09	141.2 ± 11.79	150.2 ± 15.40	**.02**	136.9 ± 12.88	139.3 ± 12.98	.51
BUN (mg/dl)	14.79 ± 5.47	16.68 ± 634	.24	16.14 ± 7.2	17.46 ± 7.42	.51	15.0 ± 5.86	16.84 ± 6.6	.28
Creatinine (mg/dl)	0.84 ± 0.19	0.93 ± 0.15	.06	0.92 ± 0.26	1.12 ± 0.35	**.01**	0.86 ± 0.22	1.14 ± 0.32	**.0005**
HbA1c (%)	6.55 ± 0.39	6.78 ± 0.46	.06	6.7 ± 057	6.85 ± 0.63	.38	6.56 ± 0.56	6.9 ± 0.63	**.04**
ALT (IU/Lit)	31.66 ± 13.24	35.63 ± 9.60	.22	31.86 ± 16.48	42.21 ± 21.72	**.03**	25.41 ± 13.83	37.24 ± 19.06	**.01**
AST (IU/Lit)	32.14 ± 13.87	36.88 ± 11.21	.18	34.17 ± 16.16	43.79 ± 16.32	**.04**	30.21 ± 13.28	41.60 ± 15.91	**.006**
ALP (IU/Lit)	94.79 ± 35.45	98.92 ± 38.82	.68	107.3 ± 42.69	107.9 ± 47.55	.96	95.52 ± 37.27	106.9 ± 43.77	**.35**
LDH (IU/Lit)	110.1 ± 36.43	125.6 ± 28.3	.08	128.8 ± 52.29	183.8 ± 62.77	**.0009**	119.0 ± 43.56	148.8 ± 28.29	**.005**
Ferritin (ng/ml)	146.5 ± 64.87	179.9 ± 69.46	.07	218.8 ± 148.3	317.5 ± 141.3	**.01**	166.2 ± 86.84	276.1 ± 137.1	**.0008**

P‐value< .05 is significant.

#### 3.3.2 Metformin users and COVID‐19 patients without T2DM

A comparison of the study parameters between metformin users and COVID‐19 patients without T2DM is shown in Table 2. As indicated, except for FBS and HbA1c, no significant differences were observed in the parameters between the mentioned groups.

**TABLE 2:** Comparison of study parameters in metformin users and healthy individuals before infection, during infection and after recovery.

Before infection				During infection			After recovery		
Variables	Metformin users	Healthy individuals	*p*‐Value	Metformin users	Healthy individuals	*p*‐Value	Metformin users	Healthy individuals	*p*‐value
FBS (mg/dl)	135.7 ± 9.30	88.43 ± 7.02	**<.0001**	141.2 ± 11.79	89.57 ± 6.62	**<.0001**	136.9 ± 12.88	88.23 ± 5.51	**<.0001**
BUN (mg/dl)	14.79 ± 5.47	14.7 ± 4.6	.94	16.14 ± 7.2	16.2 ± 4.06	.96	15.0 ± 5.86	14.93 ± 2.98	.95
Creatinine (mg/dl)	0.84 ± 0.19	0.82 ± 0.13	.55	0.92 ± 0.26	0.85 ± 0.12	.23	0.86 ± 0.22	0.93 ± 0.12	.15
HbA1c (%)	6.55 ± 0.39	5.05 ± 0.24	**<.0001**	6.7 ± 057	5.03 ± 0.21	**<.0001**	6.56 ± 0.56	4.99 ± 0.19	**<.0001**
ALT (IU/Lit)	31.66 ± 13.24	30.17 ± 11.35	.65	31.86 ± 16.48	31.21 ± 10.71	.85	25.41 ± 13.83	29.28 ± 9.36	.21
AST (IU/Lit)	32.14 ± 13.87	32.23 ± 10.47	.97	34.17 ± 16.16	33.41 ± 11.21	.83	30.21 ± 13.28	30.57 ± 9.47	.90
ALP (IU/Lit)	94.79 ± 35.45	90.27 ± 30.49	.60	107.3 ± 42.69	91.63 ± 28.32	.1	95.52 ± 37.27	87.90 ± 28.24	.32
LDH (IU/Lit)	110.1 ± 36.43	106.9 ± 25.46	.70	128.8 ± 52.29	113.4 ± 28.15	.16	119.0 ± 43.56	103.9 ± 22.27	.09
Ferritin (ng/ml)	146.5 ± 64.87	135.6 ± 42.67	.47	218.8 ± 148.3	176.1 ± 70.38	.16	166.2 ± 86.84	134.0 ± 37.02	.07

P‐value< .05 is significant.

#### 3.3.3 Metformin non‐users and COVID‐19 patients without T2DM

Before infection, FBS, HbA1c, creatinine, LDH and ferritin were higher in metformin non‐users than healthy subjects (*p*‐value: <.0001, <.0001, .005, .01 and .005, respectively). In addition, all study parameters except BUN and ALP were significantly lower in infected people without T2DM during infection. A similar pattern was repeated after recovery (Table 3).

**TABLE 3:** Comparison of study parameters in metformin non‐users and healthy individuals before infection, during infection and after recovery.

Before infection				During infection			After recovery		
Variables	Metformin non‐users	Healthy individuals	*p*‐Value	Metformin non‐users	Healthy individuals	*p*‐Value	Metformin non‐users	Healthy individuals	*p*‐Value
FBS (mg/dl)	138.5 ± 11.54	88.43 ± 7.02	**<.0001**	150.2 ± 15.40	89.57 ± 6.62	**<.0001**	139.3 ± 12.98	88.23 ± 5.51	**<.0001**
BUN (mg/dl)	16.68 ± 6.34	14.7 ± 4.6	.18	17.46 ± 7.42	16.2 ± 4.06	.42	16.84 ± 6.6	14.93 ± 2.98	.16
Creatinine (mg/dl)	0.93 ± 0.15	0.82 ± 0.13	**.005**	1.12 ± 0.35	0.85 ± 0.12	**.0002**	1.14 ± 0.32	0.93 ± 0.12	**.001**
HbA1c (%)	6.78 ± 0.46	5.05 ± 0.24	**<.0001**	6.85 ± 0.63	5.03 ± 0.21	**<.0001**	6.9 ± 0.63	4.99 ± 0.19	**<.0001**
ALT (IU/Lit)	35.63 ± 9.60	30.17 ± 11.35	.06	42.21 ± 21.72	31.21 ± 10.71	**.01**	37.24 ± 19.06	29.28 ± 9.36	**.04**
AST (IU/Lit)	36.88 ± 11.21	32.23 ± 10.47	.11	43.79 ± 16.32	33.41 ± 11.21	**.007**	41.60 ± 15.91	30.57 ± 9.47	**.002**
ALP (IU/Lit)	98.92 ± 38.82	90.27 ± 30.49	.35	107.9 ± 47.55	91.63 ± 28.32	.12	106.9 ± 43.77	87.90 ± 28.24	.057
LDH (IU/Lit)	125.6 ± 28.3	106.9 ± 25.46	**.01**	183.8 ± 62.77	113.4 ± 28.15	**<.0001**	148.8 ± 28.29	103.9 ± 22.27	**<.0001**
Ferritin (ng/ml)	179.9 ± 69.46	135.6 ± 42.67	**.005**	317.5 ± 141.3	176.1 ± 70.38	**<.0001**	276.1 ± 137.1	134.0 ± 37.02	**<.0001**

P‐value< .05 is significant.

## 4 DISCUSSION

Since the end of 2019, the SARS‐CoV‐2 infection has involved millions of people around the world. There are numerous studies indicate that the impact of this disease is worse among individuals with comorbidities like diabetes.^13^ Interestingly, better glycaemic management leads to improvement in clinical outcomes in infected people. Until now, growing evidence suggests that pre‐existing metformin treatment appears to alleviate COVID‐19 outcomes.^8^ In an observational study, it was shown that metformin reduced the mortality rate in women with T2DM.^14^ However, in randomized trial that tested the effectiveness of metformin, ivermectin and fluvoxamine in preventing severe form of COVID‐19, none of these drugs prevented the occurrence of severe form.^15^ Studies have shown several laboratory abnormalities, such as elevated liver enzymes, ferritin, D‐dimer and cardiac troponin I, mirror the severity of the COVID‐19.^16^ In the current study, we analysed the results of some biochemical tests between COVID‐19 patients with T2DM who used metformin and patients who consumed agents other than metformin to examine the effect of this drug on the severity of SARS‐CoV‐2 infection. According to our findings, FBS, creatinine, AST, ALT, LDH and ferritin were lower in metformin‐users and COVID‐19 patients without T2DM during infection. In a study conducted with Ombretta Para et al, high ferritin level was related to adverse outcomes in Italian population.^17^ Furthermore, Karanvir Kaushal et al, reported higher ferritin level in patients with thrombotic complications.^18^ In our study, ferritin decreased after recovery in metformin users and healthy individuals compared to metformin non‐users. However, Gameil et al. found high levels of inflammatory markers such as ferritin after survival.^19^ In a recent study, Huang et al. reported that elevated levels of LDH and ALT can be appropriate markers to distinguish between the severe and non‐severe forms of COVID‐19.^20^ In concert with this study, Guan et al. introduced elevated aminotransferase amounts in patients with severe disease in comparison with non‐severe individuals.^21^ In a study on hospitalized patients due to COVID‐19, the elevation of ALT–AST levels was associated with a more severe course and increased mortality in COVID‐19.^22^ Also, our results indicate that after recovery, biochemical parameters were reduced in metformin users, and except for FBS and HbA1c, no significant difference was observed between this group and healthy individuals. This is while in metformin non‐users no significant reduction was seen after recovery in compared with other groups. In a follow‐up study by Guiling Li et al, significant improvement was seen in laboratory tests such as ALT, GGT, CRP and ALP after recovery.^23^

According to our findings in all groups FBS was increased during infection and did not differ significantly after recovery. A recent study revealed that COVID‐19 infection caused an increase in blood glucose levels, even in those with no diabetes history.^24^ However, in current study no significant reduction was observed after recovery in subgroups. It is worth noting that HbA1c was reduced after recovery from COVID‐19 infection in metformin users and healthy individuals. It seems that HbA1c is slightly higher in people with severe form than those with mild form.^24^ During our study, we faced limitations such as lack of other important laboratory tests, chest CT imaging, and low sample size. Unfortunately, there was no information regarding COVID‐19 symptoms to investigate their relationship with metformin use, as Lalau et al. examined in their study.^25^ Also future studies with larger cohorts and different ethnics are required to understand the exact effect of metformin on COVID‐19 outcomes.

## 5 CONCLUSION

The current study revealed that metformin may be associated with improved COVID‐19 outcomes in patients with T2DM. Nevertheless, more research with larger sample size is required to prove the protective effect of metformin against SARS‐CoV‐2 infection.

## AUTHOR CONTRIBUTIONS

**Rana Taheri:** Data curation (equal); formal analysis (equal); investigation (equal); resources (equal); software (equal); writing – original draft (equal); writing – review and editing (equal). **Zahra shahrokhi:** Funding acquisition (equal); methodology (equal); supervision (equal); writing – review and editing (equal). **Zahra Amjadi:** Data curation (equal); writing – original draft (equal). **Faranak Kazerouni:** Conceptualization (lead); funding acquisition (lead); project administration (lead); supervision (lead); validation (lead); visualization (lead); writing – review and editing (lead).

## FUNDING INFORMATION

The present article is financially supported by Research Department of the School of Allied Medicine‐ Shahid Beheshti University of Medical Sciences.

## CONFLICT OF INTEREST STATEMENT

The authors declare that they have no conflict of interest.

## Data Availability

The data that support the findings of this study are available on request from the corresponding author. The data are not publicly available due to privacy or ethical restrictions.
